# Single Strain High-Depth NGS Reveals High rDNA (ITS-LSU) Variability in the Four Prevalent Pathogenic Species of the Genus *Candida*

**DOI:** 10.3390/microorganisms9020302

**Published:** 2021-02-02

**Authors:** Claudia Colabella, Debora Casagrande Pierantoni, Laura Corte, Luca Roscini, Angela Conti, Matteo Bassetti, Carlo Tascini, Vincent Robert, Gianluigi Cardinali

**Affiliations:** 1 Department of Pharmaceutical Sciences, University of Perugia, 06121 Perugia, Italy; c.colabella@izsum.it (C.C.); deboracasagrandepierantoni@gmail.com (D.C.P.); laura.corte@unipg.it (L.C.); roscini.lu@gmail.com (L.R.); angela.conti@studenti.unipg.it (A.C.); 2 Istituto Zooprofilattico Sperimentale dell’Umbria e delle Marche “Togo Rosati”, 06126 Perugia, Italy; 3 Department Science and Health, University of Genoa and Genoa Hospital, 16100 Genova, Italy; matteo.bassetti@unige.it; 4 Department of Medical Art, University of Udine and Udine Hospital, 33100 Udine, Italy; c.tascini@gmail.com; 5 Westerdijk Fungal Biodiversity Institute, Uppsalalaan 8, 3584 CT Utrecht, The Netherlands; vrobert@bio-aware.com; 6 CEMIN Excellence Research Centre, University of Perugia, 06123 Perugia, Italy

**Keywords:** next generation sequencing (NGS), Sanger, ITS, LSU, intragenomic rRNA variation, Maldi-Tof, *Candida albicans*, NCAC, typing, yeast

## Abstract

Ribosomal RNA in fungi is encoded by a series of genes and spacers included in a large operon present in 100 tandem repeats, normally in a single locus. The multigene nature of this locus was somehow masked by Sanger sequencing, which produces a single sequence reporting the prevalent nucleotide of each site. The introduction of next generation sequencing led to deeper knowledge of the individual sequences (reads) and therefore of the variants between the same DNA sequences located in different tandem repeats. In this framework, NGS sequencing of the rDNA region was used to elucidate the extent of intra- and inter-genomic variation at both the strain and species level. Specifically, the use of an innovative NGS technique allowed the high-throughput high-depth sequencing of the ITS1-LSU D1/D2 amplicons of 252 strains belonging to four opportunistic yeast species of the genus *Candida*. Results showed the presence of a large extent of variability among strains and species. These variants were differently distributed throughout the analyzed regions with a higher concentration within the Internally Transcribed Spacer (ITS) region, suggesting that concerted evolution was not able to totally homogenize these sequences. Both the internal variability and the SNPs between strain can be used for a deep typing of the strains and to study their ecology.

## 1. Introduction

The ribosomal DNA (rDNA) operons are typically arranged in one of more locus in arrays of tandem repeat elements comprising highly conserved sequences interspersed with more variable regions [1,2].

The question on the genetic mechanisms leading to the homogenization of multigene families like rRNA genes has been long debated—since 1972, when Brown proposed unequal crossing over as a potential explanation of the fact that copies of the same gene in the same genome seem to evolve in a concerted manner [3]. Seven years later, Jeffries proposed gene conversion as the mechanism able to clear variant copies within human globin multigene families [4], and this mechanism became the most accepted to explain the whole phenomenon. Last in the debate, the birth and death evolution was proposed as an alternative to the other two models by Nei [5]. For ribosomal DNA there is a large consensus in favor of gene conversion, although the birth and death evolution mechanism was insisted to be present in some filamentous fungi [6].

Due to the ubiquity of ribosomal RNA molecules in all cellular life forms, comparative analysis of conserved and variable rDNA regions has been universally applied to discriminate organisms at different taxonomic levels and clarify phylogenetic relationships between species and populations. For example, the 16S rDNA sequence in prokaryotes and the D1/D2 region of the large subunit (LSU) rRNA gene in yeasts are widely used in species identification and for molecular and phylogenetic reconstruction [7,8,9]. Furthermore, the internally transcribed spacer (ITS) sequence has been recently proposed as the primary DNA barcode marker for fungi, being mostly easily amplified and sequenced and providing acceptable resolution in a wide range of taxa [10], especially using well curated databases [11,12]. However, in the last few years, many studies have been pointing out some limitations in using ITS for delimiting and identifying fungal species. The most serious issues are related to the lack of resolution between closely related species [13,14,15], and more troublingly, to the presence of non-homologous copies of the ITS in the genome.

Intragenomic ITS variation is already well documented for some fungi, including some species of the genus *Candida* [16,17,18,19]. This variation may derive from recombination, gene duplication and point mutations produced by DNA replication errors and may result in erroneous identification, especially in metabarcoding studies. The correct interpretation of ITS variants, in fact, is particularly crucial in the mounting multitude of environmental metabarcoding, considering that natural and sequencing variants will produce wrong estimations of diversity and/or the introduction of “ghost” taxa. It was estimated, in fact, that the number of fungal ITS reads in the SRA (Sequence Read Archive) surpasses the number of fungal ITS sequences accessioned in GenBank by a factor of more than 10,000 [20].

In recent years, cloning has been used to unveil the variability levels [21,22], finding high variation rates among fungi. Similarly, next generation sequencing (NGS) has been applied, but with a relatively low level of coverage in most instances [23,24,25]. Results are often contrasting due to differences in the rDNA region studied, technique employed and level of coverage. The last parameter is probably critical because with over one hundred repeats [24], a coverage of at least 1000× is necessary to randomly sample each copy ten times. Lower values are likely to underestimate this internal variability.

In this framework, this study aimed to elucidate the extent of rDNA sequence variation at both the strain and species level by using high-depth NGS sequencing of the rDNA region. The study was carried out on a panel of 252 strains of the four prevalent pathogenic species of the genus *Candida*. This strain set was specifically chosen to give a reliable and representative characterization of the rRNA heterogeneity of freshly isolated strains that did not have time to undergo laboratory-induced variations. Results showed the presence of a large extent of intra- and inter-genomic variation among the strains and between the species. These variants were differently distributed throughout the regions analyzed with a higher concentration within the ITS region. The implications of our findings were discussed in the light of a good understanding of the variability of these markers for the appropriate interpretation of data, but also for the impact they may have on the diagnostics of these important medical species.

## 2. Materials and Methods

### 2.1. Strains and Growth Conditions

The 252 strains employed in this study were previously isolated from patient blood cultures in two Italian hospitals (Pisa Hospital and Udine Hospital) and extensively described in our previous paper (Table S1) [26]. Strains belong to the four prevalent pathogenic species of the genus *Candida* (*Candida albicans*, *Candida tropicalis*, *Candida parapsilosis* and *Candida glabrata*) were included in the CEMIN Microbial Collection (CMC) of the Microbial Genetics and Phylogenesis Laboratory of CEMIN (Centre of Excellence on Nanostructured Innovative Materials for Chemicals, Physical and Biomedical Applications—University of Perugia). The first step of cultivation was carried out on YEPDA medium (yeast extract 1%, peptone 1%, dextrose 1%, agar 1.7%—all products from Biolife—http://www.biolifeitaliana.it/) at 37 °C. Strains were grown in YPD medium and grown at 37 °C under shaking at 150 rpm.

### 2.2. MALDI-TOF Analysis

For MALDI-TOF analysis the selected strains were grown over night in YEPDA medium. For each strain, one colony was suspended in 300 μL of nuclease-free water. Further, 900 μL of absolute ethanol (Carlo Erba reagents—http://www.carloerbareagents.com/) was added and mixed carefully, and the samples were centrifuged (13,000× *g* for 2 min). Supernatant was discarded and each pellet was dried at 37 °C for 5 min. In the subsequent step, 20 μL of a 70% aqueous solution of formic acid and 20 μL of acetonitrile (Carlo Erba reagents) were added to the pellet, mixed and centrifuged (13,000× *g* for 2 min). The supernatant (1 μL), containing ribosomal proteins, was deposited on the 96 well MALDI plate and dried at room temperature. Samples were overlaid with 1 μL of matrix solution (Bruker Matrix HCCA α-cyano-4-hydroxycinnamic acid, Bruker Daltonik, GmbH—http://www.bruker.com/) and then dried at room temperature. For each sample two spots on MALDI plate (technical replicates) were made.

The measurements were performed with a Microflex mass spectrometer (Bruker Daltonik, Bremen, Germany) using FlexControl software (version 3.4.85.1 Bruker, Bremen, Germany). The spectra were recorded in the positive linear mode (laser frequency, 60 Hz; ion source 1 voltage, 20 kV; ion source 2 voltage, 18.24 kV; lens voltage, 0.01 kV; mass range, 2000 to 20,000 Da). For each spectrum 200 shots in 40-shot steps from different positions of the target spot (automatic mode) were collected and analyzed. The MALDI-TOF spectra were internally calibrated by using *Escherichia coli* ribosomal proteins (Bruker IVD Bacterial Test Standard).

### 2.3. DNA Extraction and Sequencing 

Genomic DNA was extracted according to the procedure suggested by Cardinali et al. (Cardinali, Bolano et al. 2001). ITS1, 5.8S, ITS2 rDNA genes and D1/D2 domain of the LSU were amplified with FIREPole^®^ Taq DNA Polymerase (Solis BioDyne, Tartu, Estonia), using ITS1 (5′-TCCGTAGGTGAACCTGCGG)-NL4 (GGTCCGTGTTTCAAGACGG) primers. The amplification protocol was carried out as follows: initial denaturation at 94 °C for 3 min, 30 amplification cycles (94 °C for 1 min, 54 °C for 1 min and 72 °C for 1 min) and a final extension at 72 °C for 5 min. Amplicons were subjected to electrophoresis on 1.5% agarose gel (Gellyphor, EuroClone, Italy). Amplicons were sequenced with paired-end (150-bp reads; NGS PlexWell™ technologies, seqWell^TM^, Beverly, MA, USA) with the same primers used for the generation of the amplicons. Library preparation, sequencing and base calling were carried out by seqWell™ (http://www.seqwell.com/).

### 2.4. Data Analysis

#### 2.4.1. MALDI-TOF Data Analysis

MALDI-TOF spectra were imported into the BioTyper software (version 3.1 Bruker, Bremen, Germany) and analyzed by standard pattern matching with default settings, using the database included in the software and regularly updated by the Bruker Company. The accuracy of the results was expressed with scores ranging from 0 to 3. Scores below 1.7 could not be considered as reliable identification, scores ≥1.7 were recognized as identification at the genus level and scores of ≥2.0 were considered useful for species identification.

#### 2.4.2. Sequencing Data Analysis

Reads of each strain, as a FASTQ file, were analyzed with Geneious R9 software (version 9.1.5, Biomatters, Auckland, New Zealand—http://www.geneious.com/). Sequencing reads were first filtered to remove reads shorter than 140 bp. The remaining reads were merged using BBMerge algorithm with default parameter settings and reads quality was assessed using the Phred quality. Identification was carried out as indicated by Colabella and colleagues [16].

#### 2.4.3. Analysis of Variant Sites

**Figure 1:**
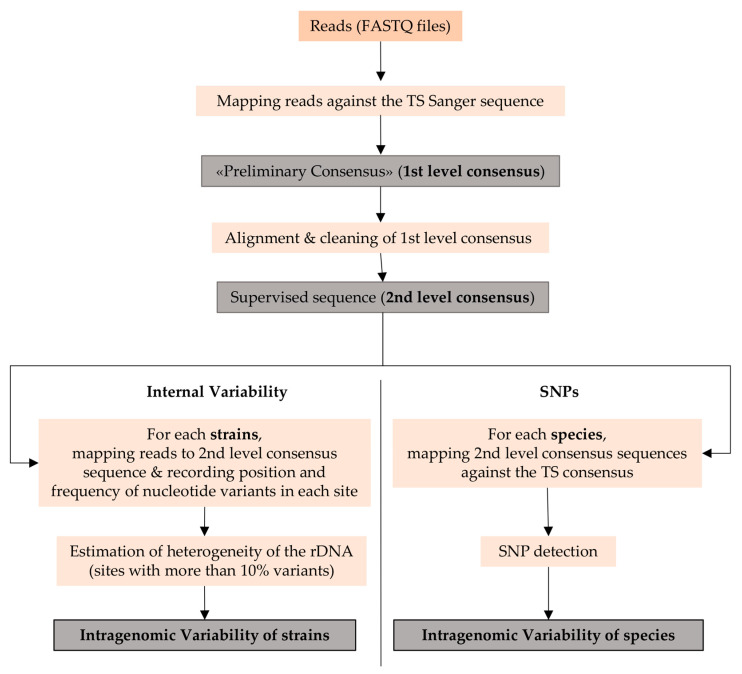
Steps in variant sites analysis.

The analysis of variant sites was carried out according to the procedure outlined in Figure 1:

Step 1. Definition of the Consensus Sequence of Each Strain

The reads in each FASTQ files were mapped against the Sanger sequences of the type strain of the species to which the strain belongs using Bowtie2 algorithm setting “local” with high sensitivity mode and no trimming of the sequences. The output was a preliminary consensus sequence named 1st level consensus. All first level consensuses of each of the four species were globally aligned against themselves, including the TS sequence, allowing visual inspection of the sequence and removal of ill-detected sites. This operation produces a clean second-level consensus, i.e., the most representative sequence of each strain, comparable to the Sanger sequence.

Step 2. Detection of the Internal Variability

All reads of each strain were mapped against the second-level consensus sequence to record position and frequency of the nucleotide variants in each site. Only sites with more than 10% variability were used in the subsequent analysis considering that variants with less variability could be due to artefacts or other technical problems. For all the variants, *p*-values were assigned considering the lowest as the representative of a real variant. The frequency of variants recorded in each variable site was calculated through all the reads covering the variant region in the contigs and called FVS (frequency of variants per site). In other words, if in a site there are 500 “A” and 250 “T”, with an “A” in the second level consensus, then the FSV will be 250/(500 + 250) = 0.33. Its average throughout the strains of each species (average frequency of variants per site—AFVS) was used to compare the internal variability at the species level.

Step 3. Detection of SNPs

All second-level consensus sequences were aligned with the consensus of the type strain of each species. SNP detection was performed as follows: Annotate&Predict function; FindVariations/SNPs with a Minimum Variant Frequency of 1%; separating annotations for each variant at a position. A csv table recording positions, variant frequency, average quality and variant p-value was exported and analyzed with a built-in macro in Microsoft Excel^®^ (Microsoft Corporation, Redmond, WA, USA).

All the second-level consensus sequences were deposited in GenBank ^®^ database.

#### 2.4.4. SNP-Based Phylogenetic Analysis

The distance analyses of the variant profiles were obtained on the basis of the SNPs detected in the multiple sequence alignments of all the second-level consensus sequences with the type strain of each species. The phylogenetic analysis was carried out in R environment (http://www.R-project.org) using the neighbor-joining method [27] from APE R-package (http://ape.mpl.ird.fr/) to reconstruct the trees.

## 3. Results

### 3.1. Heterogeneity of the rDNA within Strains of Candida Species

**Figure 2:**
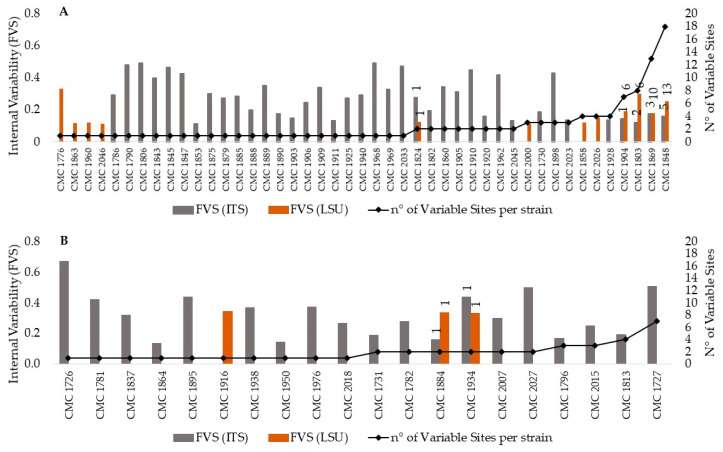
Heterogeneity of the rDNA within *Candida albicans* and *C. glabrata* strains. **Legend.** Heterogeneity of the rDNA within *C. albicans* (**A**) and *C. glabrata* (**B**) strains. Internal variability is reported on the primary y-axes as frequency of variants per site (FVS) in the internally transcribed spacer (ITS) (grey columns) and the LSU D1-D2 (orange columns) sequences of each strain. For each strain, the number of sites with more than 10% variants is reported on the secondary *y*-axis as number of variable sites. For those strains exhibiting sites with variants in both the ITS and the LSU D1-D2 sequences, the label indicates the number of variable sites detected in each region.

Forty-five out of the 161 *C. albicans* strains analyzed (around 28%) exhibited at least one site with more than 10% variants (i.e., >0.1 FSV) with respect to the second-level consensus sequence (Figure 2A). Among these, 26 strains displayed only one variable site, and the others showed from 2 to a maximum of 18 sites with variants. As expected, most of the intra-genomic variability was confined in the ITS sequences. Only five strains distributed the internal variability along both ITS and LSU D1-D2 sequences (CMC 1824, CMC 1904, CMC 1803, CMC 1869, CMC 1848), and seven strains exclusively into the LSU D1-D2 sequence (CMC 1776, CMC 1863, CMC 1960, CMC 2046, CMC 2000, CMC 1858, CMC 2026).

In order to better define the internal variability, we estimated the frequencies of variants recorded at each variant site (frequency of variants per site, FVS), with respect to the second-level consensus sequence of each strain. For the 45 strains of *C. albicans*, this value ranged from 0.11 to 0.49 (Table S2), with an average value (AFVS) of 0.28 for the ITS and 0.17 for the LSU D1-D2 region. Interestingly, the highest FVS values for the ITS sequence were detected for strains with the lowest numbers of variant sites. In fact, FVS values over 0.4 were found for the strains CMC 2033, CMC 1968, CMC 1845, CMC 1790, CMC 1847, CMC 1806 (one variable site), CMC 1910, CMC 1962 (two variable sites) and CMC 1898 (three variable sites). As the number of variable ITS sites increased, a general reduction in the average of the frequencies recorded at each site was observed. The 12 strains with intra-genomic LSU variability always presented FVS values below 0.2, regardless of the number of variant sites detected. CMC 1776, CMC 1803 and CMC 1848 strains were an exception with 0.33, 0.30 and 0.25 FVS values at 1, 6 and 13 variable sites, respectively.

NGS sequencing revealed the presence of intra-genomic variability in 20 out of the 31 *C. glabrata* strains analyzed; i.e., around 64% of the strains showed at least one site with more than 10% variants (Figure 2B). The presence of variants was limited to the ITS in 17 strains, and it involved the LSU ribosomal DNA region as well in the others. Despite the small number of variable sites detected (from 1 to 7), AFVS values were higher than those found for *C. albicans* strains—0.32 for the ITS and 0.34 for the LSU (Table S3). Moreover, six strains displayed a frequency of variants in each variable ITS site well over 0.32. Specifically, strains CMC 2027, CMC 1727, CMC 1726, CMC 1781, CMC 1895 and CMC 1934 showed FVS values of 0.50, 0.51, 0.67, 0.42, 0.44 and 0.44, respectively. Interestingly, the low variability found in the LSU sequence was restricted to one variant site per strain with about the same number of variants (0.34, 0.34 and 0.33 FVS values for CMC 1916, CMC 1884 and CMC 1934, respectively).

**Figure 3:**
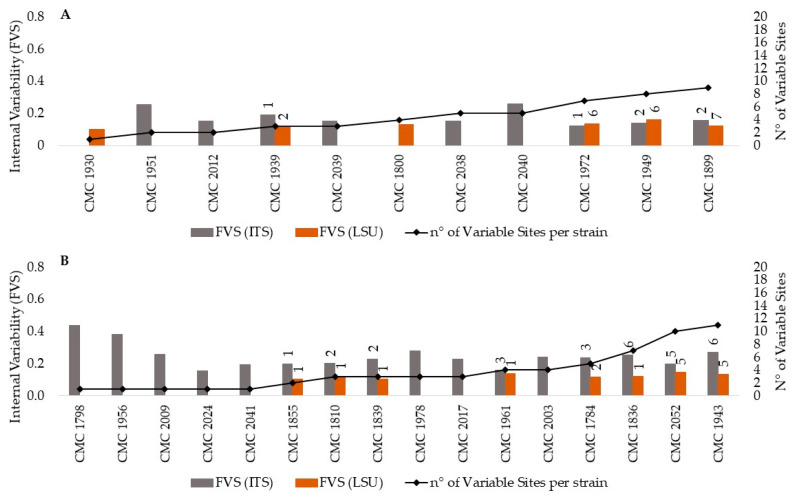
Heterogeneity of the rDNA within *C. parapsilosis* and *C. tropicalis* strains. **Legend.** Heterogeneity of the rDNA within *C. parapsilosis* (**A**) and *C. tropicalis* (**B**) strains. Internal variability is reported on the primary y-axes as frequency of variants per site (FVS) into the ITS (grey columns) and the LSU D1-D2 (orange columns) sequences of each strain. For each strain, the number of sites with more than 10% variants is reported on the secondary *y*-axis as number of variable sites. For those strains exhibiting sites with variants in both the ITS and the LSU D1-D2 sequences, the label indicates the number of variable sites detected in each region.

Overall, the intragenomic heterogeneity detected in *C. parapsilosis* and *C. tropicalis* strains was less intense than that found for strains of *C. albicans* and *C. glabrata* species (Figure 3). Specifically, only 27% of the *C. parapsilosis* strains analyzed displayed variable sites, from 1 to 5 in the ITS and from 1 to 7 in the LSU D1-D2 rDNA regions (Figure 3A). The corresponding FVS values ranged between 0.12 and 0.26 in the former and 0.1 and 0.16 in the latter, with average values (AFVS) of 0.18 and 0.15, respectively (Table S4). The intragenomic variability was distributed only in the ITS sequence in strains CMC 1951, CMC 2012, CMC 2039, CMC 2038 and CMC 2040; in the LSU region in strains CMC 1930 and CMC 1800; and in both sequences in CMC 1972, CMC 1949, CMC 1939 and CMC 1889 strains.

On the contrary, the analysis carried out on *C. tropicalis* strains showed that 16 out of the 19 strains sequenced displayed at least one site with more than 10% variants (Figure 3B). Despite this high number of strains with variant reads, the intragenomic variability was similar to that described above for *C. parapsilosis* both in terms of distribution of variant sites (1–6 sites ITS; 1-8 ITS-LSU) and of LSU AFVS values (0.15 vs. 0.15). Conversely, a greater number of variants per variable site was detected in the ITS sequences (0.26 vs. 0.18) (Table S5).

### 3.2. Internal Variability of Candida Species (SNP)

**Figure 4:**
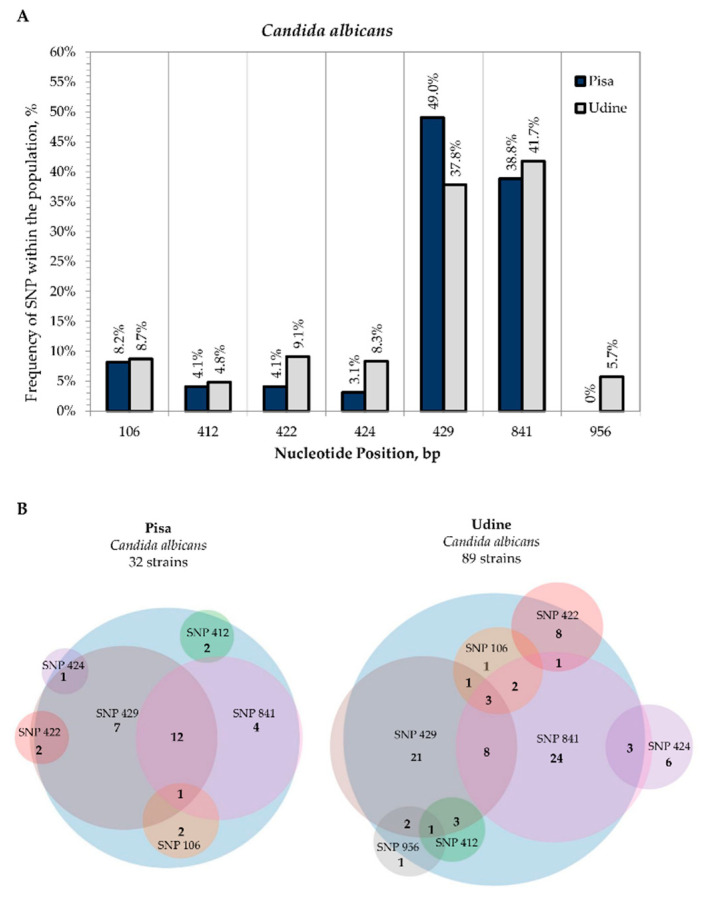
Internal variability of *C. albicans* species (SNPs). (**A**): Frequency of SNP within the population (*y*-axis) vs. nucleotide position (*x*-axis). For both sampling sites, SNP frequencies were calculated on the basis of the coverage in the multiple sequence alignment of all the second-level consensus sequences with the type strain. Frequencies of SNPs from Pisa Hospital (blue columns) and Udine Hospital (grey columns). (**B**): Venn diagrams of strains isolated from Pisa Hospital and Udine Hospital that displayed the presence of SNPs.

All second-level consensus sequences were aligned with the relative type strains to collect single nucleotide polymorphisms (SNPs). In the set of 161 *C. albicans* strains analyzed, differences between second-level consensus sequences and the *C. albicans* CBS 562 reference sequence were found to occur at seven rDNA sites for 32 strains from Pisa and 89 strains from Udine. Interestingly, all these SNPs were in the same nucleotide position regardless of the site of isolation, except for four strains isolated from Udine Hospital showing a unique SNP (5.7% frequency) in position 956 of the LSU D1-D2 sequence (Figure 4A).

Most strains showed polymorphisms at sites positions 429 of the ITS2 region (23 from Pisa; 39 from Udine) and 841 of the LSU D1-D2 domain (17 from Pisa; 41 from Udine), with frequencies of 49% and 37.8% for the former, respectively, and 38.8% and 41.7% for the latter. Among these, 12 strains from Pisa and 8 strains from Udine displayed both SNPs in positions 429 and 841 (Figure 4B). SNPs were found to occur in more than two sites in only 3.3% of strains, specifically for Pisa CMC 2023 and for Udine CMC 1879, CMC 1890 and CMC 1907 strains at nucleotide positions 106, 429 and 841 (Table S6).

**Figure 5:**
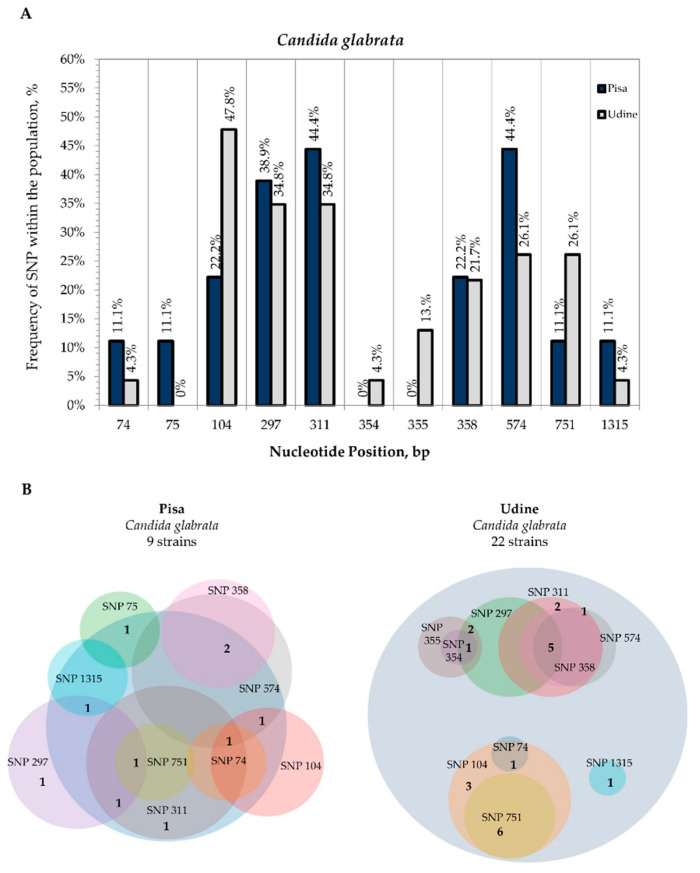
Internal variability of *C. glabrata* species (SNPs). (**A**): Frequency of SNP within the population (*y*-axis) vs. nucleotide position (*x*-axis). For both sampling sites, SNP frequencies were calculated on the basis of the coverage in the multiple sequence alignment of all the second-level consensus sequences with the type strain. Frequencies of SNPs from Pisa Hospital (blue columns) and Udine Hospital (grey columns). (**B**): Venn diagrams of strains isolated from Pisa Hospital and Udine Hospital that displayed the presence of SNPs.

In *C. glabrata* species, differences detected in the global alignment of the second-level consensus sequences with the *C. glabrata* CBS 138 reference sequence were exhibited by all 31 strains sequenced in the study. All SNPs were in the ITS region except for CMC 2027 (Pisa) and CMC 1781 (Udine) strains that showed a SNP in the LSU D1-D2 rDNA, position 1315 (Figure 5A). Four strains isolated from Pisa Hospital showed polymorphisms at site positions 311 and 574 with frequencies of 44.4%; among them only one strain showed the co-presence of these SNPs in addition to those at 74 and 104 nucleotide positions (Figure 5B). SNPs were found to occur at more than two sites only for strain CMC 2018 at positions 297, 311 and 751 (Table S8). Six out of 22 *C. glabrata* strains isolated from Udine Hospital showed the co-presence of polymorphisms at nucleotide positions 104 (47.8%) and 751 (26.1%), whereas strains CMC 1796, CMC 1813, CMC 1864, CMC 1865 and CMC 1964 displayed the presence of the same variants in the ITS positions 297, 311, 358 and 574 (Figure 5B and Table S7).

**Figure 6:**
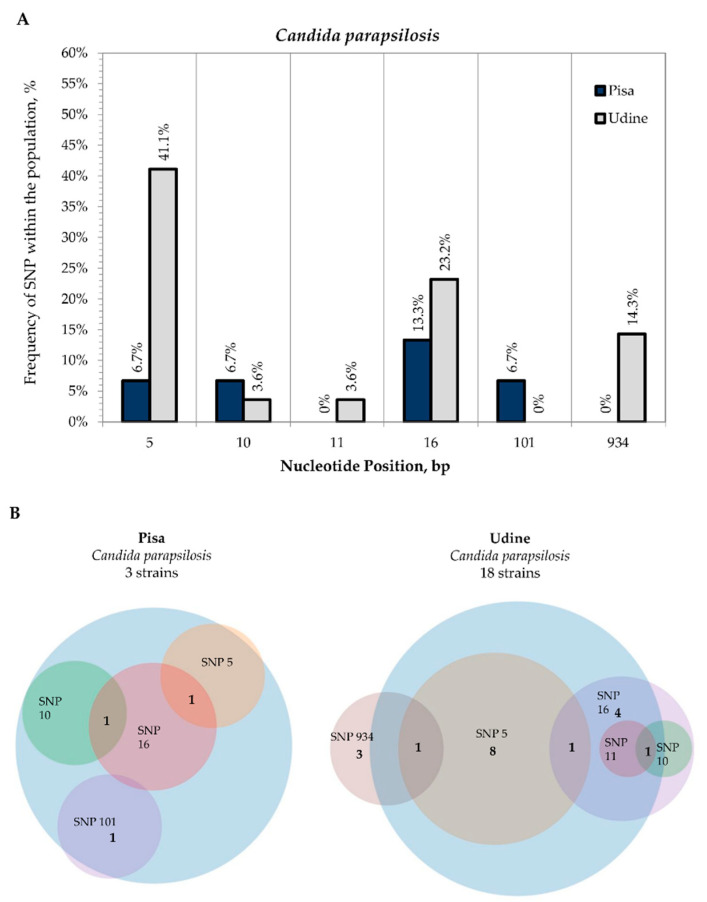
Internal variability of *C. parapsilosis* species (SNPs). **Legend.** Internal variability of *C. parapsilosis* species (SNPs). (**A**): Frequency of SNPs within the population (*y*-axis) vs. nucleotide position (*x*-axis). For both sampling sites, SNP frequencies were calculated on the basis of the coverage in the multiple sequence alignment of all the second-level consensus sequences with the type strain. Frequencies of SNPs from Pisa Hospital (blue columns) and Udine Hospital (grey columns). (**B**): Venn diagrams of strains isolated from Pisa Hospital and Udine Hospital that displayed the presence of SNPs.

The variability observed in *C. parapsilosis* species was scarce, with only six SNPs detected and most strains showing only one polymorphism with respect to the *C. parapsilosis* CBS 604 reference sequence. Specifically, among the 41 *C. parapsilosis* strains sequenced, only 3 strains from Pisa and 18 strains from Udine displayed SNPs. These polymorphisms concerned the ITS region, except for four strains isolated from Udine hospital that showed one SNP in the LSU rDNA site 934 (Figure 6A). Interestingly, most strains isolated from both Udine and Pisa hospitals exhibited the same polymorphisms in the ITS positions 5, 10 and 16. Conversely, the SNP at 11 nucleotide position of the ITS sequence was only detected for CMC 1800 from Udine, while that at 101 was only detected for CMC 2040 from Pisa Hospital (Figure 6B; Table S8).

**Figure 7:**
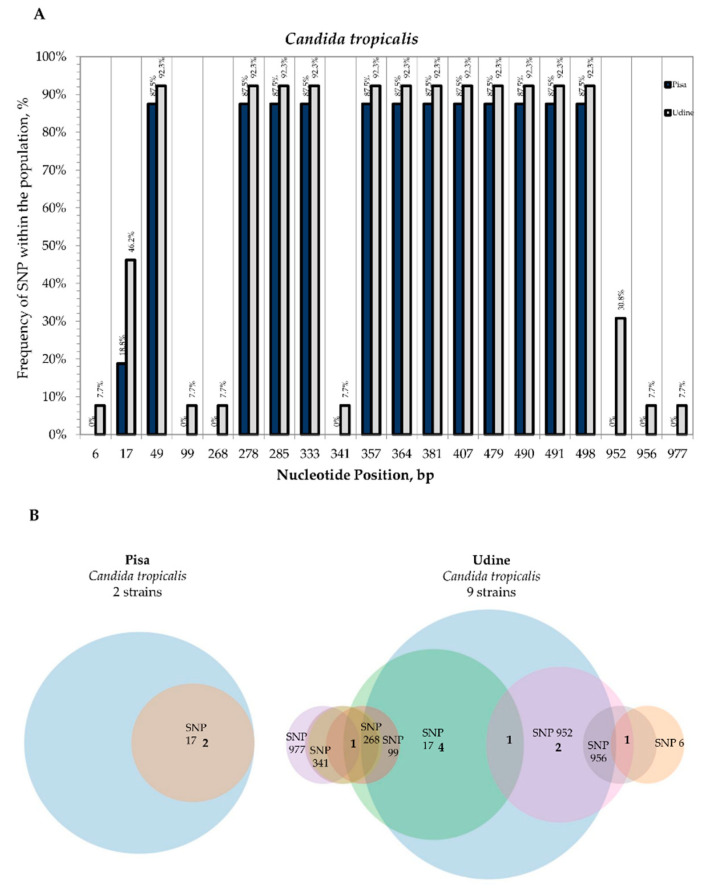
Internal variability of *C. tropicalis* species (SNPs). (**A**) Frequency of SNPs within the population (*y*-axis) vs. nucleotide position (*x*-axis). For both sampling sites, SNP frequencies were calculated on the basis of the coverage in the multiple sequence alignment of all the second-level consensus sequences with the type strain. Frequencies of SNPs from Pisa Hospital (blue columns) and Udine Hospital (grey columns). (**B**) Venn diagrams of strains isolated from Pisa Hospital and Udine Hospital that displayed the presence of SNPs not related to the type strain sequences.

Conversely, in all the 19 *C. tropicalis* strains, differences were detected in the global alignment of the second-level consensus sequences with the *C. tropicalis* CBS 94 reference sequence and revealed the presence of several polymorphisms in both the ITS and LSU D1-D2 rDNA regions. These variable sites were in the nucleotide positions 49, 278, 285, 333, 357, 364, 381, 407, 479, 490, 491 and 498 with the maximum frequencies of 87.5% and 92.3% for Pisa Hospital and Udine Hospital, respectively (Figure 7A).

Despite this large variation vs. the CBS 94 type strain, the presence of other polymorphisms was found to occur only for two strains isolated from Pisa (CMC 2009 and CMC 2024) and a few strains isolated from Udine that displayed SNPs in positions 6, 17, 99, 268, 341, 952, 956 and 977 with frequencies ranging from 7.7% to 46.2% (Figure 7A).

The presence of these polymorphisms was shown in the Venn diagrams where the *C. tropicalis* CBS 94 reference sequence was removed from the multiple alignment of the 19 second-level consensus sequences (Figure 7B). SNPs were found to occur at more than two sites for strains CMC 1855 (17, 99, 268, 341 and 977) and CMC 1943 (6, 952 and 956) isolated from Udine Hospital (Table S9).

### 3.3. SNPs-Based Phylogenetic Analysis of Candida Strains

**Figure 8:**
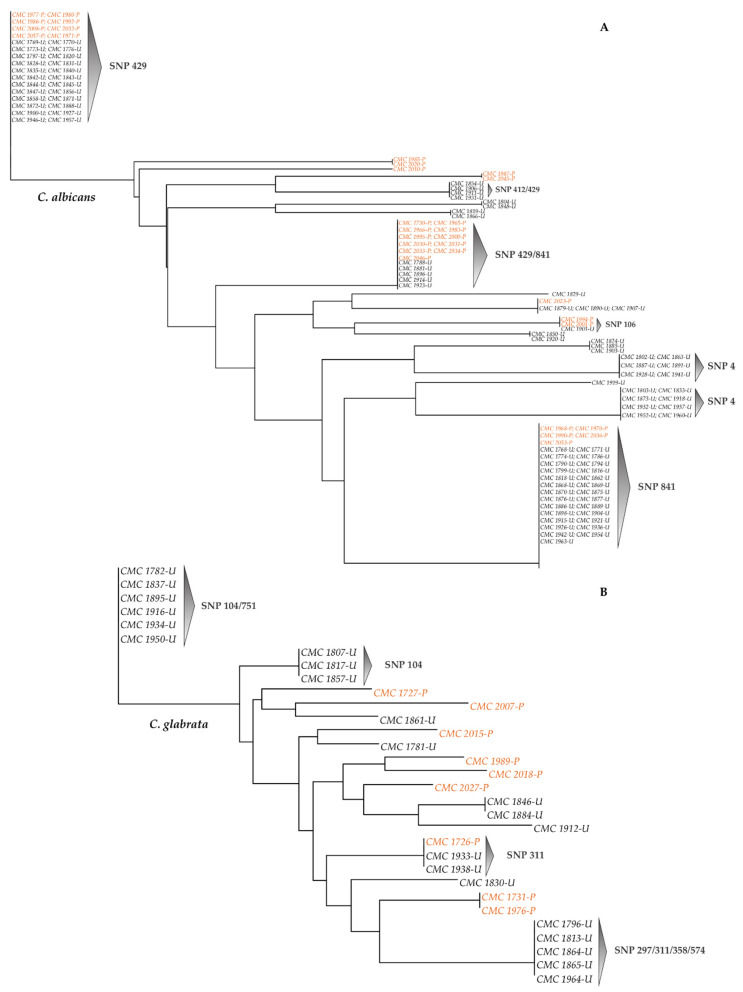
Neighbor-joining phylogenetic trees based on SNP profiles. (**A**) *C. albicans* neighbor-joining phylogenetic tree; (**B**) *C. glabrata* neighbor-joining phylogenetic tree. Black plain characters: Udine Hospital; orange plain characters: Pisa Hospital.

**Figure 9:**
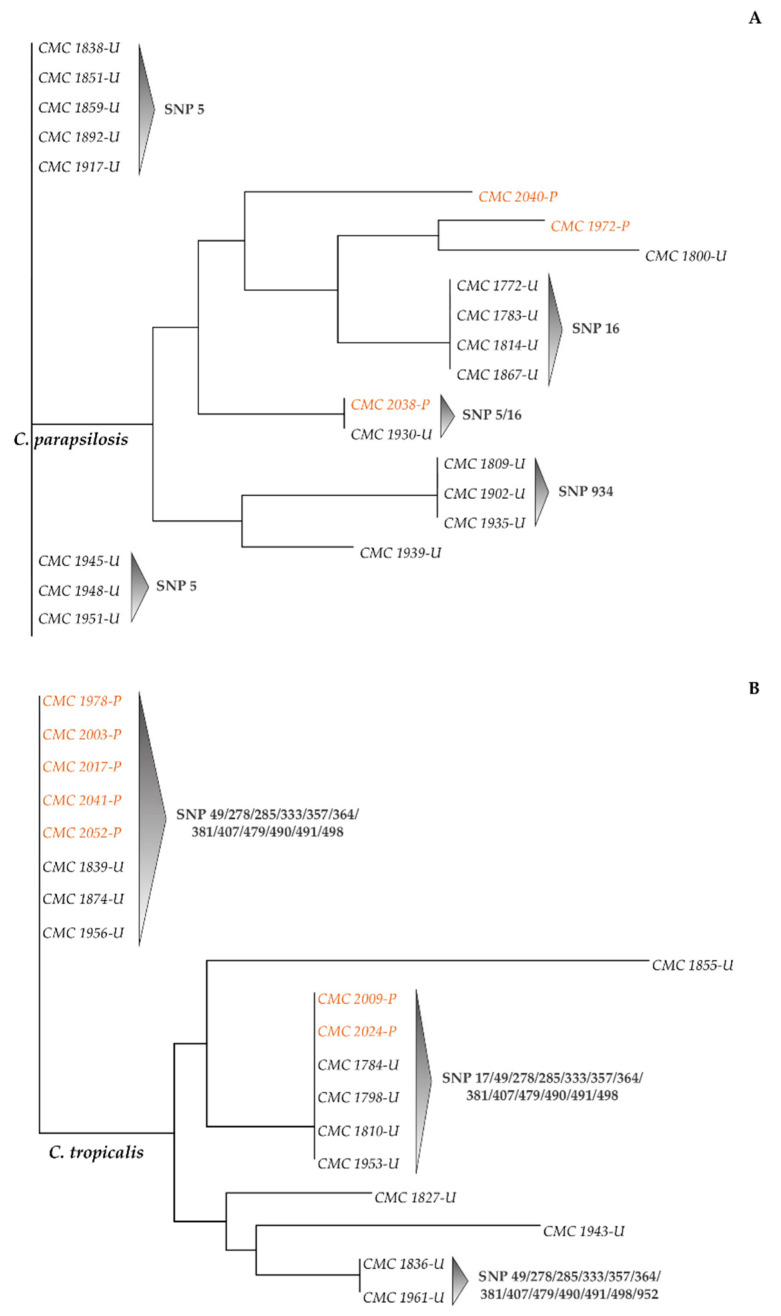
Neighbor-joining phylogenetic trees of the *C. parapsilosis* and *C. tropicalis* strain sets. (**A**) *C. parapsilosis* neighbor-joining phylogenetic tree; (**B**) *C. tropicalis* neighbor-joining phylogenetic tree. Black plain characters: Udine Hospital; orange plain characters: Pisa Hospital.

rDNA-based phylogenetic trees (Figure 8 and Figure 9) were constructed for each species based on the matrices of the rDNA SNPs detected (Tables S6–S9).

In the *C. albicans* data set, the phylogenetic tree confirmed that most of the strains isolated from both hospitals were grouped together on the basis of the polymorphisms detected from the multiple sequence alignment with the type strain reference sequence. In fact, strains were distributed into three major groups, which included the strains showing SNPs at sites 429 and 841 and sharing both (Figure 8A). Interestingly, most of the strains that showed the co-presence of SNPs at 429 and 841 sites exhibited 100% identity, regardless of the city or of the ward of isolation, suggesting the presence of strong clonality among them (Table S10). Different strains isolated from Udine Hospital formed small distinct clades related to polymorphisms at nucleotide positions 422 and 424.

The SNPs-based phylogenetic tree of the 19 *C. glabrata* strains shows a distribution of the strains isolated from Udine Hospital into three main clades, according to the Venn diagrams of Figure 5B. Strains were grouped through the base of the polymorphisms detected at sites 104 and 311 and of the shared positions 104/751 and 297/311/358/574 (Figure 8B). In the distance matrix of *C. glabrata* species the five strains isolated from Udine Hospital, recording variants in the four prevalent nucleotide positions, showed a percentage of identity of 100%. These strains also matched the same identity score for Pisa strains CMC 1731 and CMC 1976, with which they are phylogenetically related, sharing the co-presence of SNPs 358 and 574. The same percentages were also observed for those strains from Udine Hospital which had joint polymorphisms at nucleotide positions 104 and 751 and were grouped together in the phylogenetic tree (Table S11).

In the *C. parapsilosis* data set, the strains that showed differences between the second-level consensus sequence and type strain CBS 604 reference sequence were mainly those isolated from Udine Hospital (Figure 9A). These strains were grouped into three clades, according to the polymorphic sites 5, 16 and 934. Strains sharing SNPs at positions 5 and 16 were grouped in a small subclade represented by Pisa CMC 2038 and Udine CMC 1930. All the strains isolated from Udine Hospital with polymorphisms at site 5 displayed 100% identity (Table S12). Moreover, the only three strains with SNPs in the LSU position 934 (CMC 1809, CMC 1902 and CMC 1935) were identical clones. Matched scores of 100% identity were also observed among strains isolated from both hospitals that shared SNP in the ITS position 16 (i.e., CMC 2038, CMC 1772, CMC 1783, CMC 1814 and CMC 1867) and that were not grouped together by the phylogenetic analysis. Interestingly, the majority of strains from Pisa Hospital (11 out of 14) that did not display polymorphisms compared to the reference sequence were identical, showing also 100% identity among strains that did not carry SNPs isolated from “General Medicine” ward from Udine Hospital (Table S12).

As expected, strains of *C. tropicalis* were distributed into two main clades regardless of the city of isolation and according to the large extent of variability detected with respect to the CBS 94 reference sequence (Figure 9B). In fact, for all these 19 strains, the percentage of identity with the type strain reference sequence did not exceed 98.62% (Table S13). Moreover, strains with the same SNP profiles exhibited 100% identity (i.e., CMC 2017, CMC 1956 and CMC 1874). According to these findings and based on the MALDI-TOF scores (Table S1), a deeper investigation was carried out to verify if these strains had been misidentified. However, the alignment of second-level consensus sequences with type strains reference sequences of *C. albicans*, *C. glabrata* and *C. parapsilosis* (Table S14–S16) confirmed their attribution to the *C. tropicalis species*.

## 4. Discussion

Amplicon-based high throughput sequencing of rRNA encoding DNA (hereinafter rDNA) is widely used to investigate microbial communities at a scale impracticable for Sanger sequencing or culture-based methods [28,29,30,31]. However, the use of NGS technology is complemented by many technical issues, starting from the choice of the NGS platform or the difficulties in library preparation and PCR amplification [32,33]. One of the most crucial issues is how interspecific and intraspecific variation in rRNA locus may affect the analysis of microbial diversity, especially for eukaryotic microbes. In this study we investigated the extent of rDNA heterogeneity at both the strain and species level by using high-depth NGS sequencing. The study was carried out on 252 strains of the four prevalent pathogenic species of the genus Candida. The representativeness of the four species in the strain set was unbalanced in favor of *C. albicans*, represented by 161 isolated strains. This data confirmed that, despite the increasing incidence of candidiasis caused by non-*C. albicans Candida* species (NCAC), *C. albicans* remains the most common species causing infections [34,35] due to its high persistence in hospitals triggered by its superior ability to form biofilms [26,36]. No strain from another source was added to relieve the species unbalancing, because we aimed at giving a reliable and representative characterization of the rDNA heterogeneity of freshly isolated strains that did not have time to undergo laboratory-induced variations. It is in fact important to underline that all experiments were carried out with the original cultures frozen at −80 °C immediately after isolation.

Different extents of intragenomic variation were found to occur in 27% *C. parapsilosis*, 28% *C. albicans* strains and in most of *C. glabrata* and *C. tropicalis* (64% and 84%, respectively). As expected, the internal variability of the ITS sequence was greater than that of the LSU, except for *C. glabrata* strains in which the latter exceeded, even if slightly, the former (0.32 and 0.34 average frequency of variant per site for ITS and LSU, respectively). This large amount of variation at the rDNA locus does not represent a problem in single-strain NGS analysis, which, like Sanger sequencing, can mask this intragenomic variation by supplying an “average sequence” in which polymorphic sites are cut down to the most abundant nucleotide. Conversely, when reads are obtained from environmental total DNA, every read is potentially an amplicon sequence variant (ASV) that can therefore be associated with one “strain”. With some one hundred tandem repeats in each genome, even with a single read reporting enough differences, that single strain will be estimated as two strains and maybe as a member of two species. This phenomenon is prone to lead to alpha diversity overestimation and/or introducing “ghost” taxa, especially if the pipeline does not get rid of artefactual reads. These artefacts are generated randomly and are therefore expected to be non-particularly frequent. In this study we tested different threshold levels ending with 10% to ensure that less frequent variants were not taken into consideration. These contrasting aspects led to the necessity of modulating the threshold level, below which variants were not taken into consideration. Our analysis was carried out with a variant of the Illumina technology, producing short reads that were mapped to a known Sanger sequence of the type strain, as explained in the material and methods section. With this technology we are unable to predict whether all the variants were concentrated in one or few tandem repeats or were randomly scattered throughout all the repeats. In the first case, sequencing the variable repeat would likely generate an ill estimate of diversity and indicate the presence of species that indeed are not present. In the latter case, this internal variability would not produce many problems. A deep analysis of the internal variability with a long-read platform, of course in our laboratory, is expected to determine how the variants are clustered throughout the genome. In general, long-read sequencing technologies will avoid the need for contig assembly, making pipelines more fluid and avoiding the possibility that two reads from different strain are accidentally mapped together producing a sort of “chimera”. At the same time, large and curated reference databases of rDNA sequences are necessary to provide a standardized method of microbial ASVs/OTUs assignment. The use of single-copy loci [37,38,39] as targets for amplicon sequencing would effectively resolve the problem if we could identify a panel of loci with an appropriate level of sequence divergence for taxonomic assignment together with sufficiently conserved regions for an appropriate primer design.

The second aspect of this paper is that SNPs detected with NGS and purged with the procedure presented can give important clues on the strains isolated from various locales. The analysis of rDNA heterogeneity at the species level pointed out that the occurrence of SNPs is scattered throughout the whole region with a higher concentration in the ITS marker sequence.

In *C. albicans* species, SNPs was found to occur for 75% of the strains analysed only involving few rDNA sites and mostly arranged in the same nucleotide position, regardless the city of isolation. The same can be stated for *C. parapsilosis* species, for which SNPs were found for only half of the strains analysed but mostly at the same location. These data suggest strong clonality, as expected from asexual species. In addition, a careful examination of these tightly related strains revealed a close date of isolation in the same hospital, suggesting that indeed some of them can be copies of the same isolate or close relatives.

Conversely, *C. glabrata* showed the highest level of polymorphisms compared to *C. albicans* and *C. parapsilosis*. *C. glabrata* is the only species out of the four analyzed that underwent a whole genome duplication (WGD), like S. cerevisiae [40]. Moreover, *C. glabrata* has two rDNA loci [41], suggesting that the observed variation can be the sum of the variants occurring in the two loci repeats that could not be separated in our analyses. The highest variability of SNPs was observed in *C. tropicalis*, where all the polymorphisms detected were distributed in both marker sequences leading to a similarity slightly below the limit of species attribution in the distance matrix. Despite this large diversity, our analyses suggest that these strains belong to *C. tropicalis* species but certainly need to undergo further deeper studies, particularly due to their large distances from the type strains, also confirmed by MALDI-TOF outcomes.

The data presented here indicate that the intra-genomic variation can cause some ill identifications in metabarcoding studies with an obvious increase of the alpha diversity. This will happen especially if the variability is concentrated in a few tandem repeats, which is a question beyond the capabilities of the technology employed for this paper. On the other hand, the rDNA variants produced highly interesting SNPs that can help with typing the strains, their origins and their prevalence. Avoiding overestimation of the diversity and making good use of SNPs can be done with more advanced long-read platforms and with careful databases reporting SNPs and the relative metadata of the strains.

## 5. Conclusions

Amplicon-based high throughput sequencing of rDNA is widely used to investigate microbial communities at scales impracticable for Sanger sequencing or culture-based methods. However, the use of NGS technology is accompanied by many technical issues, including the choice of the NGS platform and the difficulties in library preparation and PCR amplification. One of the most critical issues is the variability of multigene families within the same strain as the rDNA repeats. In a genomic context, in which the DNA is known to derive from the same strain, this internal variability gives clues on the internal evolution in contrast with the homogenization mechanisms described in the introduction. When the same technology is applied to metabarcoding, the internal variability can be confused with the inter- and intraspecific variation, leading to ill identifications and overestimation of the alpha diversity. At the same time, a deeper analysis of these loci produces high resolution typing that allows one to detect population structure, and the presence and prevalence of the strains. As in many other cases in science, a new technology produces problems and gives new opportunities.

## Data Availability

The data presented in this study are available upon request from the corresponding author.

## Supplementary Materials

The following are available online at https://www.mdpi.com/2076-2607/9/2/302/s1. Table S1. Strains employed in the study. For each strain were reported: the GenBank accession numbers; the strain number (CMC); the scores from MALDI-TOF identification; the biofilm forming ability (biofilm forming, BF; not-biofilm forming, NBF); the ward of isolation and the city of isolation (Pisa, P; Udine, U). Key for the ward of isolation: Specialist Medicine (SP); Surgery (S); Intensive Care Unit (ICU), General Medicine (GM); Oncohematology (O); Rehabilitation (R). Table S2. Internal variability of *C. albicans* strains. For each strain were reported: the city of isolation (Pisa, P; Udine, U), the strain number (CMC), the frequencies of variants recorded at each variable ITS or LSU D1-D2 sites, the number of variable sites per ITS and LSU D1-D2 sequence, the frequency of variants per ITS and LSU site (FVS), the maximum variability per variable site and the average frequency of variants per ITS and LSU site (AFVS). Table S3. Internal variability of *C. glabrata* strains. For each strain were reported: the city of isolation (Pisa, P; Udine, U), the strain number (CMC), the frequencies of variants recorded at each variable ITS or LSU D1-D2 sites, the number of variable sites per ITS and LSU D1-D2 sequence, the frequency of variants per ITS and LSU site (FVS), the maximum variability per variable site and the average frequency of variants per ITS and LSU site (AFVS). Table S4. Internal variability of *C. parapsilosis* strains. For each strain were reported: the city of isolation (Pisa, P; Udine, U), the strain number (CMC), the frequencies of variants recorded at each variable ITS or LSU D1-D2 sites, the number of variable sites per ITS and LSU D1-D2 sequence, the frequency of variants per ITS and LSU site (FVS), the maximum variability per variable site and the average frequency of variants per ITS and LSU site (AFVS). Table S5. Internal variability of *C. tropicalis* strains. For each strain were reported: the city of isolation (Pisa, P; Udine, U), the strain number (CMC), the frequencies of variants recorded at each variable ITS or LSU D1-D2 sites, the number of variable sites per ITS and LSU D1-D2 sequence, the frequency of variants per ITS and LSU site (FVS), the maximum variability per variable site and the average frequency of variants per ITS and LSU site (AFVS). Table S6. Internal variability of *C. albicans* species (SNP). For each strain were reported: the city of isolation (Pisa, P; Udine, U), the strain number (CMC), the number of SNPs detected at each nucleotide position of ITS and LSU D1-D2 rDNA regions. Table S7. Internal variability of *C. glabrata* species (SNP). For each strain were reported: the city of isolation (Pisa, P; Udine, U), the strain number (CMC), the number of SNPs detected at each nucleotide position of ITS and LSU D1-D2 rDNA regions. Table S8. Internal variability of *C. parapsilosis* species (SNP). For each strain were reported: the city of isolation (Pisa, P; Udine, U), the strain number (CMC), the number of SNPs detected at each nucleotide position of ITS and LSU D1-D2 rDNA regions. Table S9. Internal variability of *C. tropicalis* species (SNP). For each strain were reported: the city of isolation (Pisa, P; Udine, U), the strain number (CMC), the number of SNPs detected at each nucleotide position of ITS and LSU D1-D2 rDNA regions. Table S10. Distance matrix of *C. albicans* species. For each strain were reported: the city of isolation (Pisa, P; Udine, U), the strain number (CMC) and the percentages of identity among strains. Table S11. Distance matrix of *C. glabrata* species. For each strain were reported: the city of isolation (Pisa, P; Udine, U), the strain number (CMC) and the percentages of identity among strains. Table S12. Distance matrix of *C. parapsilosis* species. For each strain were reported: the city of isolation (Pisa, P; Udine, U), the strain number (CMC) and the percentages of identity among strains. Table S13. Distance matrix of *C. tropicalis* species. For each strain were reported: the city of isolation (Pisa, P; Udine, U), the strain number (CMC) and the percentages of identity among strains. Table S14. Distance matrix of *C. tropicalis* species compared to *C. albicans* type strain. For each strain were reported: the city of isolation (Pisa, P; Udine, U), the strain number (CMC) and the percentages of identity among strains. Table S15. Distance matrix of *C. tropicalis* species compared to *C. glabrata* type strain. For each strain were reported: the city of isolation (Pisa, P; Udine, U), the strain number (CMC) and the percentages of identity among strains. Table S16. Distance matrix of *C. tropicalis* species compared to *C. parapsilosis* type strain. For each strain were reported: the city of isolation (Pisa, P; Udine, U), the strain number (CMC) and the percentages of identity among strains.
